# Color Fixation Strategies on Sustainable Poly-Butylene Succinate Using Biobased Itaconic Acid

**DOI:** 10.3390/polym13010079

**Published:** 2020-12-28

**Authors:** Lidia G. Quiles, Julio Vidal, Francesca Luzi, Franco Dominici, Ángel Fernández Cuello, Pere Castell

**Affiliations:** 1 Tecnopackaging, Polígono Industrial Empresarium C/Romero Nº, 12, 50720 Zaragoza, Spain; 2 Fundación Aitiip, Polígono Industrial Empresarium C/Romero Nº, 12, 50720 Zaragoza, Spain; julio.vidal@aitiip.com; 3 Department of Civil and Environmental Engineering, University of Perugia, 05100 Terni, Italy; francesca.luzi@unipg.it (F.L.); franco.dominici@unipg.it (F.D.); 4 Escuela de Ingeniería y Arquitectura, University of Zaragoza, Av. María de Luna, 3, 50018 Zaragoza, Spain; afernan@unizar.es

**Keywords:** biopolymers, biocomposites, polybutylene succinate, itaconic acid, zirconium oxide, colorant, hardness, color fixing, gloss, aging effect

## Abstract

Biopo-lybutylene succinate (bioPBS) is gaining attention in the biodegradable polymer market due to its promising properties, such as high biodegradability and processing versatility, representing a potential sustainable replacement for fossil-based commodities. However, there is still a need to enhance its properties for certain applications, with aesthetical and mechanical properties being a challenge. The aim of the present work is to improve these properties by adding selected additives that will confer bioPBS with comparable properties to that of current counterparts such as polypropylene (PP) for specific applications in the automotive and household appliances sectors. A total of thirteen materials have been studied and compared, being twelve biocomposites containing combinations of three different additives: a commercial red colorant, itaconic acid (IA) to enhance color fixation and zirconia (ZrO_2_) nanoparticles to maintain at least native PBS mechanical properties. The results show that the combination of IA and the coloring agent tends to slightly yellowish the blend due to the absorbance spectra of IA and also to modify the gloss due to the formation of IA nanocrystals that affects light scattering. In addition, for low amounts of IA (4 wt %), Young’s Modulus seems to be kept while elongation at break is even raised. Unexpectedly, a strong aging affect was found after four weeks. IA increases the hydrophilic behavior of the samples and thus seems to accelerate the hydrolization of the matrix, which is accompanied by an accused disaggregation of phases and an overall softening and rigidization effect. The addition of low amounts of ZrO_2_ (2 wt %) seems to provide the desired effect for hardening the surface while almost not affecting the other properties; however, higher amounts tends to form aggregates saturating the compounds. As a conclusion, IA might be a good candidate for color fixing in biobased polymers.

## 1. Introduction

Technical biopolymers are becoming increasingly attractive as sustainable and good-performing polymeric materials [1]. One of the most promising biopolymers is biopo-lybutylene succinate (bioPBS). BioPBS is an aliphatic polyester synthesized from the polymerization of two biobased building blocks: succinic acid (or dimethyl succinate) and 1,4-butanediol [2,3], all coming from renewable sources. The materials and products derived from bioPBS are soft, flexible and have become a promising replacement for commodities [4] such as polyethylene terephthalate (PET), polypropylene (PP) and polyethylene (PE) as they exhibit nearly comparable mechanical properties [5] to these synthetic plastics for several applications such as packaging, construction applications, houseware, furniture or agriculture [6]. In addition, bioPBS undergoes biodegradation during disposal in compost, moist soil, fresh water and seawater [7], which reduces its potential environmental impact and makes it a promising candidate to enhance the sustainability of plastic products—as currently demanded by the markets and consumers.

However, a general drawback of biopolymers, among them bioPBS, is that they present poor aesthetic appearance compared to oil-based counterparts. Every thermoplastic material has its own innate color. A thermoplastic material in its innate color state is referred to as natural. The natural color of biopolymers usually vary from yellowish, brownish to crude whitish, that many times is not the preference of the consumer. Thus, the use of pigments or additives to tailor the appearance of biopolymers is required. In terms of color, bioPBS has a whitish bright matrix. The need to introduce these materials in the market requires the improvement of such properties, allowing customization of the final color.

The term “colorant or coloring agent” denotes a series of colored substances that affect a material’s appearance. Therefore, the introduction of a coloring agent would increase the attractiveness of the bioplastic and the potential acceptance of the material in more applications. Nevertheless, how the material responds to the colorant is a critical aspect of the overall appearance that also involves gloss and texture. Gloss is used to describe the manner in which a surface reflects light: specular reflection (shiny), diffuse reflection or it can absorb the light (dull). Regarding texture, smooth surfaces reflect light in the specular direction, whereas diffuse reflection dominates in the case of rough surfaces.

Two kinds of coloring agents are usually used to color plastics: pigments and dyes. Pigments may be either organic or inorganic in structure and are insoluble both during processing of the plastics and in the end product [8,9]. Dyes, on the other hand, are organic molecules that dissolve into the substrate to which they are applied. Azo colorants are the most important class of synthetic dyes and pigments, representing 60–80% of all organic colorants [10]. These colorants contain one or more nitrogen–nitrogen double bonds (–N = N–) in their chemical structure and may possess other functional groups [11]. They have excellent coloring properties, mainly in the yellow to red range, as well as good lightfastness. Azo colorants are used widely in substrates such as textile fibers, leather, plastics, papers, hair, mineral oils, waxes, foodstuffs, rubbers and paints.

The colorant not only needs to match the desired color but it should also satisfy other constraints such as to be chemically compatible with the base polymer matrix and to be chemically stable. In order to be able to provide a wide palette of colors, it is important to reach a good color fixation and gloss, making it long-lasting (durable), and that it also does not affect other functional key properties such as hardness or mechanical performance. Other factors influencing color strength are particle size and dispersion in the plastic matrix.

Ideally, when a biobased material is conceived, the colorants and additives used in the formulation should come from a bio-based origin [12,13]. Natural pigments and dyes present poor color fastness and yield compared to synthetic ones [14]. For this reason, it is necessary to introduce a linking agent that enhances compatibility with the polymeric matrix. Most of the natural dyes present hydroxylic groups (–OH) in their structure and for example can be esterified with polycarboxylic acids such as citric acid or itaconic acid (IA) [15]. These, and others such as amino groups, are considered color helpers. They are known as auxochromes [16], which are able to alter both the intensity and the wavelength of absorbed light influencing the physical-chemical properties of the material while they do not produce color by themselves. These are often used as color fixation chemicals enhancing the overall stability of the colored part.

Among the different linking agents used in polymer science, IA has demonstrated good performance in oil-based polymers [17]. Today, IA is exclusively produced by fermentation with carbohydrates by filamentous fungi, mainly *Aspergillus terreus* [18]. Thus, it has the potential to be produced exclusively from biomass. IA is a fully sustainable industrial building block, an ionic hydrophilic co-monomer, with a myriad of chemical applications due to its structural similarity to acrylic and methacrylic acids [17]. In fact, it presents a viable solution to replace acrylic acid in biodegradable polymers. For example, it is used in the production of lubricants, active agents, dyes, plastics, chemical fibers, etc. [19]. Other promising uses are unsaturated polyester resins, phosphate-free detergents, and in the food industry [20]. It is stable at acidic, neutral and middle basic conditions at moderate temperatures, so it was considered a good candidate for the extrusion-compounding process in which high temperatures and shear forces are achieved.

However, as it is a component usually used in the formulation of gums/elastomers and latex, one of the drawbacks of using IA in different thermoplastics is the softening effect, which might be accentuated by the organic nature of the diazo pigment. The use of inorganic fillers has been demonstrated to be a good strategy in composites for enhancing mechanical properties [21,22,23,24,25]. Among these fillers, nanoparticles such as ZrO_2_ have been used in several applications [26,27]. Its natural whitish color, and its excellent dimensional stability, mechanical and chemical properties, has made zirconia a highly attractive ceramic material in medical applications such as hip head replacement instead of titanium or alumina prostheses and in particular for prosthodontics [28,29]. Zirconia has also been used to produce hard coatings for plastic surfaces with antifogging, anti-wetting and antistatic properties [30]. Due to this reason, it was decided to add zirconium dioxide to harden bioPBS surface as well as prevent it from losing mechanical properties.

The objective of this study is to develop an enhanced biobased material showing good aesthetical properties in terms of color fixing while keeping at least the mechanical properties of the original matrix polymer (bioPBS) in order to make it attractive for a plethora of applications in the market such as automotive, householding or furniture. In this research, IA has been selected as functional color helper to better fix and increase the lightfastness of an organic red diazo pigments in bioPBS matrix. To enhance the resulting material mechanical properties ZrO_2_ was as a reinforcing agent. A complete and detailed characterization has been conducted using micro and macroscopical techniques such as SEM, wettability, color change, hardness, and mechanical analysis. Additionally, an aging effect was evaluated during the realization of the experimental work and their effects were characterized and compared.

## 2. Materials and Methods

### 2.1. Materials

The polymer matrix used was a biobased polybutylene succinate BioPBS FZ71 PD which was purchased from Japan Pulp and Paper GmbH (Düsseldorf, Germany).

The IA 99% pure, was purchased from Sigma Aldrich (Darmstadt, Germany). It is a white crystalline powder, unsaturated dicarboxylic acid (C_5_H_6_O_4_), in which one carboxyl group is conjugated to the methylene group [31]. It presents a hygroscopic property, and it is odor-free [32,33]. Its melting point is 167–168 °C and the boiling point is 268 °C [34].

The coloring agent was purchased from UNNOX GROUPS (Esquiroz, Spain) which is an organic red diazo pigment with the commercial code: PR57:1, MDN-1153.

Finally, the Zirconia nanoparticles were kindly provided by the TORRECID group (L’Alcora, Spain). Zirconia is a crystalline dioxide of zirconium. Its mechanical properties are very similar to those of metals (it has been called ‘ceramic steel’ [35]). Zirconia crystals can be organized in three different patterns: monoclinic (found at room temperature, under ambient pressure and upon heating up to 1170 °C), tetragonal (between 1170 and 2370 °C), and cubic (above 2370 °C and up to the melting point) [36].

### 2.2. Nano-Bio-Composites Preparation

Twelve different formulations were prepared by extrusion-compounding with a 26-mm twin-screw Coperion ZSK 26 compounder machine (Stutgart, Germany). Firstly, binary blends were extruded: F2 and F3; F4 and F5 and F6. The three additives were introduced in a powder format from a secondary feeder different from the bioPBS main hopper. IA was introduced at the beginning of the barrel while the ZrO_2_ and the coloring agent at the middle of the barrel. The melted polymer and powders were mixed at a screw speed of 200 rpm; temperature was in-creased from 160 °C in the feeding zone up to 180 °C at the nozzle. The compounding was extruded through a 2 mm diameter die for a constant output of 15 kg/h. The extrudate was quenched in a water bath at room temperature, dried and cut into pellets. A total of 3 kg of per blend were produced. Ternary and quaternary formulations were produced by re-extruding in a second step using the conditions above. To this end, the colorant and the zirconia were added into bioPBS/IA extruded matrices, by introducing them at the middle of the barrel as for the first extrusion-compounding step. No modifications were required regarding the parameters and conditions of the extrusion process. Table 1 summarizes the composition of all references. 

Samples were analyzed at week 0 (W0), just after their preparation and after aging in ambient conditions at week 4 (W4).

### 2.3. General Characterisation Methods

#### 2.3.1. Two Types of Specimens Were Developed for Material Characterization

- Injected specimens for mechanical (tensile or dog-bone following ISO 178 standard) and hardness (parallelepiped specimens of 80 × 100 × 4 mm) testing were obtained by injection molding with a JSW 85 EL II electric injection machine.

The temperature profile was increased from 150 °C at the hopper up to 180 °C at the nozzle with 40 rpm. The dosage and filling pressure were varied for each formulation injected. A packing pressure of 35% (55 bar during 20 s) was applied. When injecting samples containing IA, temperatures were decreased from 140 °C at the hopper up to 160 °C at the nozzle and cooling time increased by 10 s so that it was cooled enough to be expelled from the mold.

- Circular specimens of 50 mm diameter and 2 mm thick from all the formulations were produced for measuring wettability and color change. The same processing parameters were considered. The materials were mixed for 120 s at 90 rpm in a co-rotating twin-screw extruder Microcompounder at 5 and 15 cc, DSM (Sittard, The Netherlands), using a temperature profile of 120–125–130 °C. Due to the low viscosity of the formulations containing IA, a pressure-time injection molding profile of 1.0–5; 1.1–15; 1.1–15 in bar-seconds was used. The mold and injection temperatures were set at 30 and 150 °C, respectively.

#### 2.3.2. Measurements

Mechanical tests were conducted under ambient conditions using a Zwick Roell Z 2.5 (Ulm, Germany). At least five specimens per material were tested, according to ISO 178 and ISO 527 Methodology

Structural properties were evaluated by scanning electron microscopy (SEM) with a Hi-tachi S3400N (Tokyo, Japan) equipment in order to determine the morphology and dispersion. Broken samples coming from the mechanical tests were used.

Hardness was measured using a portable hardness tester, the METALTEST tester model T500 (Barcelona, Spain), to verify material hardness with load-cell technology for Vickers, Brinell and Rockwell testing. The Rockwell B scale was used for this study.

The surface properties of produced materials were evaluated by static contact angle measurements (FTA1000 Analyzer (Newark, NJ, USA)). The wettability of the surfaces was studied by using the sessile drop method, in air, in contact with HPLC grade water. When a surface is hydrophilic, the drop extends over the material at an angle between 0° and 30°. If the surface of the solid is hydrophobic, the contact angle will be greater than 90°. On surfaces that are very hydrophobic, the angle can be greater than 150° and even close to 180°.

Color change and gloss of samples was investigated by means of a spectrophotometer (CM-2300d Konica Minolta, Japan). Data were acquired by using the SCI 10/D65 method, CIELAB color variables, as defined by the Commission Internationale de 1′Éclairage (CIE 1995), were used.

Samples were placed on a white standard plate and L*, a*, and b* parameters were de-termined. L* value ranges from 0 (black) to 100 (white); a* value ranges from −80 (green) to 100 (red); and b* value ranges from −80 (blue) to 70 (yellow). For each sample, 3 measurements were taken at random location. The total color difference or distance between colors (Euclidean distance) ∆E* between white and the samples was calculated as indicated in Equation (1) [37]: color-difference formula:

(1) $$\Delta E*=\sqrt{{\left(\Delta L*\right)}^{2}+{\left(\Delta a*\right)}^{2}+{\left(\Delta b*\right)}^{2}}$$

## 3. Results and Discussion

### 3.1. Mechanical Tests

**Figure 1:**
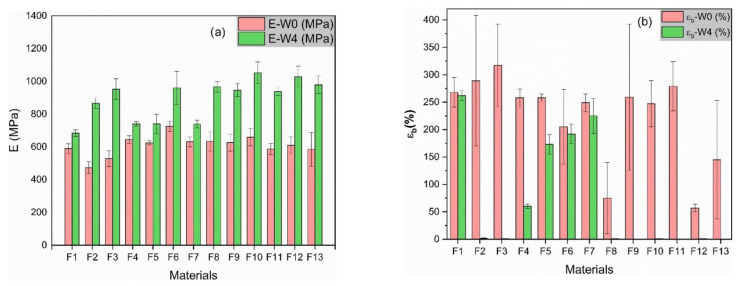
Young’s moduli (**a**) and elongation at break values (**b**) for all specimens evaluated at W0 and W4.

The mechanical properties of biocomposite materials are always a compromise between stiffness and toughness which are generally mutually exclusive. The elastic modulus (E) and elongation at break (ε_B_) are useful parameters to describe the mechanical behavior of the developed materials and are closely related to the internal microstructure. The mechanical properties determined from uniaxial tensile tests are summarized in Table 2 and their com-parison among the different formulations and aged samples are shown in Figure 1.

#### 3.1.1. Comparative Results among Formulations at W0 (Comparisons among Pink Columns—Young′s Modulus; and among Green Columns—Elongation at Break)

When IA is added to neat PBS a reduction in the Young’s modulus by 11% for F2 and by 26% for F3 is observed, while an increase in the elongation at break by 8% for F2 and by 18% for F3 is found. Thus, IA seems to induce a plasticizing effect, which is in coherence with Kirimura et al. [34] who explained the use of this component in rubber-like polymers due to its excellent strength and flexibility, making the PBS tougher. This result is also aligned with Krishnan et al. [38], who used IA to design an aliphatic copolyester elastomer that was used as PLA toughener.

The addition of the colorant (F4) as well as low amounts of ZrO_2_ (F5) slightly increases the Young’s modulus, while the material stiffness is considerable raised by 23% when a 4 wt % of ZrO_2_ is incorporated (F6). On the one hand, Zirconia affects in the elongation at break and an overall fall can be observed (F6 decreases a 24%). Nevertheless, the data dispersion is broad, that indicates the appearance of agglomerates producing a stress concentrating effect favoring a premature rupture [39]. All in all, this decrease is noticeably found for 4 wt % of ZrO_2_, which is a quite positive result for such a high content of zirconium if compared with other similar studies using it as reinforcement agent in polymers. For example, Mishra T.K. et al. [40] found the same decrease in the elongation at break for PEEK/ZrO2 compounds with just a 1 wt %. On the other hand, when the colorant is incorporated to PBS (F4) elongation at break values remain almost unchanged. Consequently, the colorant appears to be better dispersed and integrated than the ZrO_2_ probably due to its organic nature which is more compatible with the blend.

Ternary formulations (F5, F6, F8, F9, F10, F11) and quaternary blends (F12, F14), lead to a complex mechanical behavior in which the rigidity of the materials is kept or even scarcely in-creased when compared to neat PBS. The combination of these additives induce a remarkable fall in the elongation at break respect to neat PBS. The most prominent decrease is found for F8 by 72% and F12 by 79%, closely followed by F13. Both formulations are complex blends combining a 4% wt % of IA and colorant. The high scattering of the data confirms a lack of integration between the IA, the matrix and the additives, probably forming regions of high and low IA concentration peaks; and the formation of zirconia and colorant aggregates with insufficient dispersion (a result that has been corroborated with SEM micrographs).

#### 3.1.2. Comparative Results of Same Formulations between W0 and W4 (Aging Effect)

All plastic materials suffer from aging with time. They tend to recrystallize as the polymer chains end their ordering. The plastic gets stiffer and in a long-term becomes brittle. Kimble et al. [41], studied aging in PLLA/PBS blends including annealing and creep studies. As conclusion, blends with high content of PBS tend to decrease its Mw with time produced by degradation and showing and embrittlement of samples. Thus, their results also highlighted the importance of appropriate storage conditions for ductility retention. In this study, the Young’s modulus of neat PBS increased by 16% (F1) after 4 weeks. The elongation at break was maintained, that indicated the good integrity of the material and, apparently, an absence of degradation.

The effect of the IA was remarkable. In F2 and F3 samples, the Young’s modulus raised by 80%, which clearly indicated the rigidization effect. The blends lost their toughness and became very brittle. A drop in the elongation at break by more than 98% was observed in all the formulations containing IA.

When adding the colorant (F4) and a 2 wt % of ZrO_2_ (F5), the material became a 15% stiffer in both cases, while when increasing the addition of ZrO_2_ by 4 wt % (F6) the Young’s modulus doubled by reaching a 32% higher value. Stiffness and toughness are usually compromised properties, and we can find a reduction in elongation at break by 6% for F6 and by 33% for F5. These results draw a better dispersion of ZrO_2_ for F6 rather than F5. The colorant also seems to highly affect this property, falling by 77% (F4), that may indicate a disaggregation of the organic phases with time that weakens the interphase between the colorant and the additive.

When ternary and quaternary blends are studied (F8–F13), we can observe a rise in the Young’s modulus between 60% and 70%, as expected, and practically an absence of elongation at break effect induced by the IA.

### 3.2. Hardness

#### 3.2.1. Hardness Results at W0

The highest value on hardness is found for F2 (4 wt % IA), which multiplies almost by four the hardness value for neat PBS, while for F3 (10 wt %) it decreases a 18% down neat PBS value. It is likely that a 10 wt % IA has saturated the blend, showing a lack of miscibility among the two phases (polybutylene succinate (PBS) and IA). The shear forces or thermodynamics produced during the compounding process might not have been enough to disperse and homogenize the components, which seem not to be compatible and which tend to form separated phases inside the blend. Moreover, as these phases are distinct, the IA apparently seems to crystallize into the surface of the injected specimen. Using the SEM micrographs we can observe the IA crystals, and when the indenter finds a high concentration of IA in the surface it also finds a fragile and brittle point.

When observing F4, the PBS phase mainly contains the colorant, which is a soft pigment, and the overall hardness of the blend decreases by 58%. On the other hand. when characterizing F5 and F6, we can find a hardening effect by 6% for the 2 wt % of zirconia. However, when adding a 4 wt % of zirconia, hardness decreases by 31%, probably due to a saturation of the blend accompanied by the apparition of agglomerates. The high surface energy of nanoparticles is prone to induce the formation of nanoparticles aggregation [42]. Thus, phase separation often takes place owing to the great differences in the properties of polymer and inorganic materials.

When combining the colorant and the ZrO_2_ (F7), there is a negative synergistic effect, decreasing the hardness value by 82%, while the combination of any of them with the 4 wt % IA induces a positive synergy in which the IA dominates the behavior of the complex blends (F8 by 68%, F10 by 181% and F12 by 65%). However, with a 10 wt % IA added instead, it still dominates the blend keeping similar values to those found for F3.

#### 3.2.2. Hardness Results at W4

When hardness was measured at W4, the authors found a clear embrittlement of the samples. Especially in those containing IA (F2, F3 and F8–F13), to which it was not possible to repeat the characterization test. The specimens broke when the indenter penetrated the sample, and some of the experiment samples even had a ductile fracture just from being manipulated.

### 3.3. Structural Properties (SEM)

**Figure 2:**
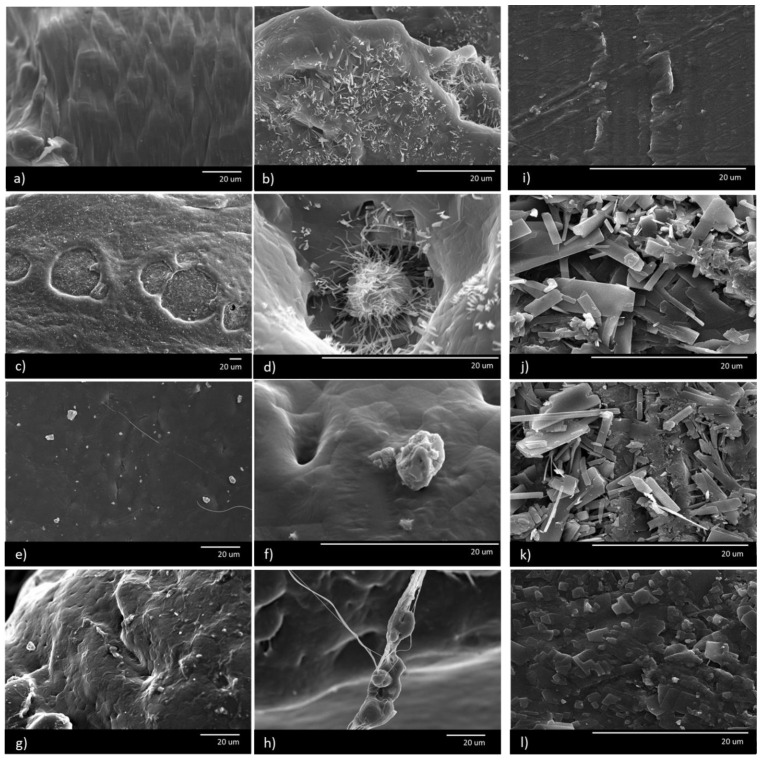
SEM—W0 (left—(**a**,**c**,**e**,**g**)—and central—(**b**,**d**,**f**,**h**)—columns) and W4 (right column—**i**,**j**,**k**,**l**). (**a**): F1; (**b**): F3; (**c**): F9; (**d**): F13; (**e**,**f**): F5; (**g**,**h**): F4; (**i**):F1; (**j**): F3; (**k**): F9; (**l**) F10.

The surface morphology of the developed formulations was studied by using scanning electron microscope (SEM). Micrographs of representative specimens are shown in Figure 2.

#### 3.3.1. Structural Results at W0

The micrographs confirm the tendencies observed in the mechanical and hardness results. IA crystallizes in the form of rectangular nanocrystals. It clearly manifests a special phase morphology constituted by the disperse phase of IA. These nanocrystals are found to group themselves in a heterogeneous way showing a lack of compatibility between the IA and the PBS (b,c). The IA nanocrystals are found to migrate to the surface of the part. The authors observed this phenomena: either over time outside to the external surface (Figure 2j,k are taken from the border perimeter), or within internal created surfaces such as for example in bubbles or defects generated during the injection process of the specimen (Figure 2d). Therefore, we find a highly reinforced material at the surface level, but as it is not miscible, it is not well integrated, and a segregation of the phases is observed.

Apparently, as the pigment is highly organic, it displays a good interfacial bonding with the polymer (Figure 2g,h). The ZrO_2_ seems to be well dispersed, although a few aggregates can be found (Figure 2e,f). When ternary and quaternary blends are studied, those containing IA nanocrystals tend to surround the colorant aggregates. This effect can be explained as probably being due to the combination of the polar–apolar performance of both materials, that generates a complex interface inducing a low adherence with the polymeric matrix, PBS. The IA is an acid, that means that the hydrogen on the OH group can easily be removed, leaving an anion. Both the colorant pigment as well as ZrO_2_, may act as a salt to the IA, favoring the attraction between them (Figure 2d). These aggregates have a stress concentrating effect which reduces the toughness of the material, favoring a premature breaking, that explains the mechanical behavior found.

#### 3.3.2. Structural Results at W4

Finally, a change in the type of fracture can be observed between W0 and W4. At W0 the fracture surface evidenced a ductile behavior (Figure 2a), while at W4 a more fragile surface is found showing a flat fracture surface of the polymer accompanied by some striations (Figure 2i), in which polymeric chains have had the time to rearrange. So does the IA, which is unstable in the blend and migrates to the surface of the specimen, seeking the state of minimum energy (Figure 2j,k,l). Thus, it can be confirmed that the materials suffer from aging that induces a rigidization mechanism and that it is clearly intensified with the addition of IA.

### 3.4. Hydrophobicity/Wettability

Wettability results are included in Table 2. The range WCA values for our bioPBS systems vary between 44° and 76°.

#### 3.4.1. Structural Results at W0

The presence of IA in binary systems reduces WCA when compared with neat PBS (F1), this phenomenon is more pronounced in F2 than in F3 making the material more hydrophilic. It is also found a slight reduction in the WCA when colorant is added (F4) while the presence of ZrO_2_ does not change the WCA value of reference PBS matrix (F5, F6). Mizuno et al. [5], showed the same tendency as with IA when introduced grafted Acrylic Acid into PBS to study and control the biodegradability of this aliphatic polyester. Their study demonstrated that a more hydrophilic surface had a considerable impact in the biodegradation of the PBS and thus it negatively affects the mechanical and structural properties.

In the samples where IA was combined with ZrO_2_ (F10, F11 systems), the WCA seems to be influenced only by the presence of IA and the contact angle values are similar to the values recorded for PBS/IA binary systems.

The addition of ZrO_2_ (F7) does not change the values of F4, influenced by the colorant pigment, as already noted in the case of ternary systems. The presence of IA reduces the values of F4, so the decrease in wettability in the presence of IA is confirmed even in colored samples (F8, F9). Moreover, as explained, the colored samples suffer from cleavage of the labile groups of the red pigment, thus accelerating even more this degradation process that is also translated in a more hydrophilic surface.

Including the ZrO_2_ in the F12 and F13 systems only slightly changes the WCA values, when compared to ternary systems having IA but without ZrO_2_ (F8, F9).

#### 3.4.2. Structural Results at W4

An overall decrease on WCA was observed comparing the values obtained for PBS samples at W0 and at W4, making the matrix material more hydrophilic with time as a result of material degradation. According to Mizuno et al., wettability together with the stereochemistry, the flexibility of molecular chains and the crystallinity, have been found to be decisive factors in the biodegradability of PBS [42]. Our results show the natural effect of conjugating the natural biodegradation process of PBS synergistically speed up by the acid transfer of IA to the PBS matrix.

### 3.5. Color Properties

Color properties of all formulations are presented in Table 3.

#### 3.5.1. Color Results at W0

On the one hand, IA (F2, F3), reduces the parameter *a* (a bit greener) and it also induces an increase in parameter *b* (more yellow), so the samples tend to have yellowish coloration (shift to warm tones). The absorbance range for red color in the UV-VIS spectra is about the 600 to 700 nm [43], while the natural absorbance peak for IA locates at 200–205 nm [44]. Therefore, the combination tends to move the overall material spectra to the lower wavelength absorbance spectra, moving down first to yellowish and then to greenish values. The gloss of the PBS–IA binary systems decreases as the IA concentration increases—probably due to the formation of nanocrystals which scatter the light in a number of different directions than the original matrix.

On the other hand, the presence of ZrO_2_ in PBS (F5, F6) causes an increase in L*, a* and b* parameters, due to its opaque white color. It can be observed that ZrO_2_ whitens the specimens and decreases also the gloss of the material. This drop in gloss values may be produced by the roughening effect induced in the matrix structure by the ZrO_2_ agglomerates.

For the ternary systems F10 and F11, that combine IA and ZrO_2_, the *L* parameter increases when compared to F2 and F3 (IA based). Again, this effect may be produced because of the presence of ZrO_2_. The gloss of F10 and F11 systems does not change if compared to binary F5 and F6 (ZrO_2_ binary systems) and F2 and F3 formulations. Thus, we can confirm that there is no sum of the effects of fillers in terms of gloss.

The presence of the 4 wt % colorant determines a considerable variation of the color parameters induced by the red color of the pigment. The combined species formed in the samples absorbs and attenuates the light [45]. The *L* parameter decreases when compared to the value recorded for PBS (that makes darker the material), a* and b* increases as expected following the red color scale. The addition of this red pigment does not change the values of the gloss (F4). The presence of ZrO_2_ in F7, F8 and F12 samples causes *L* and a parameters to increase while *b* to decrease (bluish) respect to F4, which we consider now as the sample of control. The presence of IA and ZrO_2_ in the F12 and F13 system reduces gloss, and no combined effect of the two fillers is revealed.

#### 3.5.2. Color Results at W4

After 4 weeks a considerable reduction of gloss parameter was recorded in all PBS based samples containing IA. The same behavior was also found when colorant was added. Moreover, a synergistic effect appeared when both additives are combined. The azo bond, which is known to be the most labile portion of an azo colorant, can readily undergo cleavage photochemical degradation [46]. Both, the effect of the cleavage in the polymer structure and the formation and re-grouping of IA nanocrystals vary the manner in which the surface reflects light. The internal structure of the material becomes rougher and gloss is dominated by a diffuse reflection.

For PBS samples with ZrO_2_ (F5 and F6), the gloss tends to increase with time. However, any combination of ZrO_2_ either with IA or colorant or both, is dominated by the other two, obtaining fully dull gloss samples.

## 4. Conclusions

The present work demonstrates an industrial technology to produce biobased composites with enhanced coloring properties through extrusion-compounding of complex blends. A methodology was proposed to design a biobased material with enhanced and ad hoc aesthetical properties while maintaining the natural polymer performance. To this end, a diazo red colorant has been added to a bioPBS matrix. In order to increase the color fixing, IA has been used with the aim of acting as compatibility agent between the matrix and the pigment, as its structure is quite similar to acrylic acid (we were looking for a behavior similar to paint formulations). Lastly, as IA was also modifying other fundamental properties of bioPBS, ZrO_2_ nanoparticles which have a similar natural color as the bioPBS matrix, were incorporated to avoid decreasing stiffness and hardness. An aging effect was observed during the realization of the experimental work and their effects were characterized and compared (week 0 and week 4).

Regarding the mechanical properties, at W0 the addition of the colorant and low amounts of ZrO_2_ makes the material stiffer, while it almost does not affect the elongation at break, making the material tough and ductile. The addition of IA provokes the desired effect as it maintains or even increases the Young’s modulus (26% for 10 wt % IA) while increasing the elongation at break (18% for 10 wt % IA). Concerning hardness, it is reduced by the addition of the diazo pigment, as expected, while it is augmented by the zirconia at low loads. Combinations of colorant and ZrO_2_ are found to produce a negative synergistic effect, decreasing by 82% the hardness value. In contrast, for low amounts of IA, the hardness value is multiplied almost by four times, while higher amounts tend to soft the blend. Complex blends combining the colorant, ZrO_2_ and IA shows a dominant behavior induced by IA.

At W4, a prominent embrittlement is found due to an aging effect. A drop in the elongation at break by more than 98% can be observed in all the formulations containing IA, while the Young’s modulus rises by 60% to 70% showing a rigidization effect. This behavior is accompanied by a softening of all samples being outstanding for those blends containing IA, losing their plastic deformation capacity. The loss in mechanical and hardness properties was corroborated by SEM and wettability results. When SEM micrographs were studied, IA nanocrystals were found to group themselves in a heterogeneous way showing a lack of compatibility between the IA and the matrix and a clear separation of the phases. In addition, the WCA results showed a tendency for the material to become more hydrophilic when IA was incorporated, inducing biodegradability (hydrolyzing effect with the water of the environment) and thus accelerating the degradation of the material.

Our findings on color fixing show that, on the one hand, IA has an absorbance range somewhat lower than the red spectrum, so the material tends to slightly yellow the matrix; and, on the other hand, the high formation of IA nanocrystals shown by SEM micrographs produces such a variation in the structure of the material that it modifies the way of scattering and absorbing light and decreases the gloss of the blend which is translated into a more matte finish. This effect is accentuated with time. ZrO_2_ by itself increments the gloss of the material with time, but in ternary systems IA governs the overall behavior.

To sum up, the use of IA might be a good candidate to be used as a color fixation agent. However, coupling techniques such as the use of reactive polymers (grafting to with, i.e., peroxide initiators) or the use of compatibilizers (grafting from with, i.e., amphiphilic structures) of IA with the PBS matrix should be further explored as these could favor the compatibility of the materials, therefore increasing their miscibility and avoiding the quick undesirable aging effect found on the materials (and avoiding the separation of phases and accelerated biodegradation), which would also affect the color fastening and keep the gloss. These coupling techniques may be industrialized through adapted extrusion-compounding processes, e.g., reactive extrusion (REX). Moreover, due to the natural absorbance ranges of IA, a recommended strategy is for it to be used for yellow to blue colorant pigments.

## Figures and Tables

**Table 1:** Summary of material formulations based on bioPBS FZ71PD matrix.

Reference	bioPBS Matrix	IA	Colorant	ZrO_2_
F1	100%	-	-	-
F2	96%	4%	-	-
F3	90%	10%	-	-
F4	96%	-	4%	-
F5	98%	-	-	2%
F6	96%	-	-	4%
F7	94%	-	4%	2%
F8	92%	4%	4%	-
F9	86%	10%	4%	-
F10	94%	4%	-	2%
F11	88%	10%	-	2%
F12	90%	4%	4%	2%
F13	84%	10%	4%	2%

**Table 2:** Mechanical properties under tensile force: Young’s modulus and elongation at break for all characterized materials; surface hardness and contact angles measurement (wettability). Measurements done at week 0 (W0) and after 4 weeks aging (W4).

Material	Young′s Modulus (MPa) W0	Young′s Modulus (MPa) W4	Elongation at Break (%) W0	Elongation at Break (%) W4	Hardness (HRB) W0	Hardness (HRB) W4	WCA (°) W0	WCA (°) W4
F1	590 ± 30	685 ± 20	268 ± 27	262 ± 9	6.2 ± 1.3	5.1 ± 0.9	76 ± 1	69 ± 3
F2	473 ± 37	866 ± 33	289 ± 119	1.5 ± 0.8	20.6 ± 2.6	Break	54 ± 3	54 ± 0
F3	528 ± 49	953 ± 63	317 ± 75	0.67 ± 0.05	5.1 ± 0.4	Break	66 ± 3	47 ± 3
F4	645 ± 24	740 ± 14	258 ± 16	60 ± 4	2.6 ± 1.4	2 ± 1.3	68 ± 3	70 ± 3
F5	625 ± 13	740 ± 60	258 ± 7	173 ± 18	6.6 ± 1.1	1.6 ± 0.5	75 ± 3	66 ± 1
F6	726 ± 31	960 ± 351	205 ± 68	192 ± 18	4.3 ± 1.3	1.1 ± 0.6	76 ± 3	71 ± 2
F7	631 ± 31	739 ± 23	249 ± 16	225 ± 32	1.1 ± 0.8	1.1 ± 0.8	70 ± 3	66 ± 3
F8	634 ± 59	966 ± 32	75 ± 65	0.88 ± 0.2	10.4 ± 1.5	Break	63 ± 4	56 ± 3
F9	626 ± 52	946 ± 39	259 ± 133	0.27 ± 0.01	6.4 ± 0.7	Break	65 ± 3	58 ± 4
F10	659 ± 52	1052 ± 67	247 ± 42	0.97 ± 0,4	17.4 ± 0.3	Break	59 ± 2	44 ± 3
F11	587 ± 34	937 ± 24	279 ± 45	0.56 ± 0.28	5.0 ± 1.2	Break	67 ± 2	61 ± 3
F12	610 ± 52	1027 ± 66	57 ± 7	0.88 ± 0.2	10.2 ± 1.6	Break	60 ± 3	61 ± 3
F13	585 ± 103	979 ± 53	145 ± 108	0.3 ± 0.05	5.6 ± 0.6	Break	60 ± 4	59 ± 3

**Table 3:** Color coordinates and gloss of polybutylene succinate (PBS) based systems at W0 and W4.

Material Formulations	L*	a*	b*	∆E*	Gloss (°)
White Control	99.47 ± 0.00	−0.08 ± 0.01	−0.08 ± 0.01	-	121 ± 0
F1–W0	85.80 ± 0.26	−1.22 ± 0.03	−1.04 ± 0.08	13.75 ± 0.26	78 ± 4
F1–W4	86.05 ± 0.43	−1.05 ± 0.03	−1.60 ± 0.08	13.54 ± 0.43	72 ± 1
F2–W0	86.84 ± 0.13	−1.34 ± 0.04	3.53 ± 0.08	13.20 ± 0.13	72 ± 2
F2–W4	87.34 ± 0.23	−1.07 ± 0.02	3.02 ± 0.19	12.56 ± 0.25	53 ± 3
F3–W0	84.01 ± 0.06	−2.27 ± 0.02	9.92 ± 0.10	18.54 ± 0.06	63 ± 2
F3–W4	84.37 ± 0.21	−1.68 ± 0.14	8.08 ± 0.06	17.23 ± 0.23	17 ± 2
F4–W0	35.09 ± 0.09	36.71 ± 0.26	16.06 ± 0.12	75.89 ± 0.08	77 ± 3
F4–W4	34.84 ± 0.12	35.72 ± 0.17	15.39 ± 0.15	75.49 ± 0.18	77 ± 4
F5–W0	86.43 ± 0.07	−0.41 ± 0.03	8.97 ± 0.05	15.87 ± 0.04	70 ± 2
F5–W4	86.50 ± 0.15	−0.46 ± 0.01	8.41 ± 0.13	15.51 ± 0.06	71 ± 4
F6–W0	88.34 ± 0.06	0.77 ± 0.03	13.29 ± 0.12	17.42 ± 0.09	68 ± 3
F6–W4	88.41 ± 0.10	0.73 ± 0.05	13.07 ± 0.10	17.20 ± 0.11	75 ± 2
F7–W0	39.30 ± 0.39	44.58 ± 0.12	12.51 ± 0.20	75.99 ± 0.30	66 ± 3
F7–W4	39.01 ± 0.23	43.73 ± 0.17	11.60 ± 0.27	75.57 ± 0.32	60 ± 3
F8–W0	36.18 ± 0.09	40.28 ± 0.65	15.7 ± 0.24	76.72 ± 0.38	66 ± 3
F8–W4	36.04 ± 0.36	40.81 ± 0.99	15.43 ± 0.22	77.05 ± 0.40	8 ± 2
F9–W0	34.88 ± 0.14	38.34 ± 0.38	15.39 ± 0.33	76.73 ± 0.20	66 ± 3
F9–W4	34.78 ± 0.51	39.33 ± 1.03	15.38 ± 0.41	77.32 ± 1.03	11 ± 2
F10–W0	87.62 ± 0.34	−0.56 ± 0.04	11.55 ± 0.20	16.62 ± 0.25	71 ± 3
F10–W4	88.05 ± 0.13	−0.78 ± 0.04	10.83 ± 0.40	15.81 ± 0.33	26 ± 1
F11–W0	85.44 ± 0.26	−0.46 ± 0.02	11.38 ± 0.07	18.12 ± 0.19	61 ± 3
F11–W4	85.82 ± 0.10	−0.44 ± 0.05	10.56 ± 0.10	17.31 ± 0.13	29 ± 3
F12–W0	38.25 ± 0.60	40.30 ± 0.13	12.70 ± 0.28	74.44 ± 0.52	63 ± 3
F12–W4	37.84 ± 0.52	42.11 ± 0.46	12.83 ± 0.13	75.79 ± 0.42	5 ± 1
F13–W0	38.88 ± 0.11	42.90 ± 0.11	12.98 ± 0.11	75.42 ± 0.11	67 ± 2
F13–W4	38.89 ± 0.29	44.20 ± 0.21	13.03 ± 0.20	76.17 ± 0.34	8 ± 1
